# Gap Measurement Method for Railway Switch Machines Based on the Fusion of Deep Vision and Geometric Features

**DOI:** 10.3390/s26113280

**Published:** 2026-05-22

**Authors:** Wenxuan Zhi, Qingsheng Feng, Shuai Xiao, Xilong He, Haowei Liu, Yiyang Zou, Hong Li

**Affiliations:** 1 School of Electrical Engineering, Dalian Jiaotong University, Dalian 116028, China; zwx_djtu@163.com (W.Z.); fqs@djtu.edu.cn (Q.F.); xiaos1002@126.com (S.X.); hxl152400@163.com (X.H.); jackieliu520619@163.com (H.L.); 18840838965@163.com (Y.Z.); 2 Baotou Electric Power Section, China Railway Hohhot Group Co., Ltd., Baotou 014040, China; 3 School of Mechanical Engineering, Dalian Jiaotong University, Dalian 116028, China; 4 School of Railway Intelligent Engineering, Dalian Jiaotong University, Dalian 116028, China

**Keywords:** railway switch machine, gap measurement, fusion of vision and geometric features, sub-pixel precision

## Abstract

The gap dimension of a railway switch machine is a critical physical quantity for determining the locking status of railway turnouts. Under operating conditions characterized by heavy oil contamination, complex illumination, and equipment vibration, existing visual measurement methods often struggle to maintain stability and achieve sub-pixel precision. To address this issue, this paper proposes a gap measurement method based on the fusion of vision and geometric features (G-VFM). The method first utilizes a confidence-aware optimized YOLOv8 model to achieve robust localization of the gap region. Subsequently, an improved multi-channel U-Net is employed to extract soft-edge probability maps, based on which a 20-dimensional structured geometric descriptor is constructed. Finally, visual semantic features and geometric priors are fused for regression through an R34-Fusion two-stream residual network, and systematic errors are corrected using a weighted Huber loss combined with a piecewise linear calibration strategy. Test results on a constructed field dataset show that the proposed method achieves a Mean Absolute Error (MAE) of 0.0076 mm and a maximum error of 0.0193 mm. It achieves a 100% pass rate under an industrial tolerance of 0.02 mm, with an end-to-end inference time of 52.23 ms (~19.15 FPS), balancing both precision and efficiency. Further tests on illumination degradation, noise interference, and cross-batch evaluations indicate that the method maintains relatively stable performance across various complex scenarios. However, performance decreases significantly under extremely low-light conditions, suggesting that actual deployment may require integration with active lighting or multi-sensor fusion to ensure system reliability across all working conditions. Overall, this method achieves high-precision gap measurement under current experimental conditions and provides a feasible solution for vision-based switch machine status monitoring.

## 1. Introduction

As critical equipment for route conversion in railway transit, the operational status of railway switch machines is directly related to traffic safety [1,2,3]. Under high-speed and heavy-haul conditions, the intense dynamic coupling between wheels and rails—such as wheel rail vibration and impact contact forces—is a significant factor inducing equipment performance degradation [4,5]. Recent studies have shown that vibration energy caused by wheel rail lateral instability can propagate along turnout sections, thereby inducing switch rail displacement deviations or local mechanical wear [6,7,8]. Such mechanical deformations triggered by external excitations are further transmitted to the internal locking mechanism of the switch machine. Taking the widely used ZD6 electric switch machine as an example, the physical gap between its indication rod check block and check column (i.e., the standard clearance of 1.5 ± 0.5 mm) is the essential physical basis for determining the locking state of the equipment [9]. Once the gap dimension exceeds the tolerance range, it may lead to severe consequences such as derailment. Therefore, achieving high-frequency and high-precision automated monitoring of the gap has become an important demand in intelligent railway operation and maintenance [10].

Currently, switch machine status monitoring mainly relies on periodic manual inspections or indirect inferences based on 1D time-series signals such as operating current and power curves [11,12,13,14]. Although some progress has been made in intelligent fault diagnosis and mechanism-inspired signal analysis for rotating machinery [15,16], traditional electrical signal methods typically only reflect macroscopic anomalies in the motor or hydraulic system, making it difficult to perform direct, quantitative, and intuitive evaluations of millimeter-scale mechanical gaps [14,17]. To break through the limitations of these indirect diagnostic methods and achieve direct perception of micro-gaps, non-contact measurement technology based on machine vision has gradually gained attention due to its strong spatial resolution capabilities [18]. Early studies mostly attempted to combine Canny or Sobel operators or MobileNet with clustering algorithms for gap segmentation [1,18,19,20]. However, heavy oil contamination, dust, and non-uniform illumination are prevalent in real monitoring environments. These factors easily cause edge localization failure in algorithms relying on hand-crafted features, making it difficult to guarantee monitoring robustness under complex field conditions.

Deep learning technology, represented by Convolutional Neural Networks, provides a new methodological path for complex industrial vision tasks [21,22,23,24,25]. In the railway field, YOLO architectures incorporating attention mechanisms and lightweight segmentation networks have been used for high-precision qualitative identification of rail defects and turnout anomalies [26,27,28,29,30,31]. However, most existing deep vision frameworks are designed for classification or coarse segmentation tasks [32,33]. For precision measurement tasks of switch machine gaps with an error tolerance below 0.02 mm, pure vision models still face significant physical mapping challenges. The downsampling process of the network causes the loss of high-frequency spatial information, leading to blurring at the edges of predicted masks; furthermore, there is a lack of a rigid constraint mechanism to stably map pixel coordinates to absolute physical dimensions [34,35].

To transcend the limitations of physical image resolution, sub-pixel edge detection technology has been widely applied in precision engineering [36]. Traditional methods usually rely on Zernike moments or spline interpolation to fit the grayscale distribution [37,38,39,40], while recent studies have begun to attempt combining deep features with geometry-driven frameworks to improve noise resistance [34,35,41]. Inspired by this, this paper proposes a ZD6 switch machine gap measurement method, G-VFM, based on the fusion of deep vision and geometric features. This method jointly models deep semantic vision with explicitly constructed structured geometric priors during the regression stage to alleviate the bias issues of pure vision methods under complex interference conditions, thereby enhancing the model’s ability to represent micro-scale changes. The main work of this paper can be summarized as follows:

1. A cascaded visual processing workflow consisting of ROI localization and edge perception is constructed to achieve stable key region extraction and edge representation under complex background and illumination disturbance conditions;
2. A vision and geometric feature fusion modeling mechanism is designed. By introducing structured geometric descriptors into the regression process, the model gains certain physical constraints during learning, thereby improving fitting stability at the sub-pixel scale;
3. The measurement precision, stability, and cross-batch consistency of the method were verified on the constructed dataset and experimental conditions. The results show that the method can reach the expected precision level in the current scenario and maintain relatively stable performance within a certain range.

## 2. Experimental Acquisition System and Dataset Construction

The robustness of a deep learning measurement framework is highly dependent on the quality of the physical representation of the input data. Given the current lack of publicly available sub-pixel high-fidelity image datasets in the field of railway switch machines, and considering that conventional data struggles to cover severe operating conditions such as heavy oil contamination, high-frequency vibration, and extreme illumination in real industrial sites, this paper first established a physical acquisition system close to industrial sites before unfolding the specific algorithmic network design. Furthermore, a multi-condition dataset oriented toward micro-gap measurement was independently constructed. This chapter will elaborate on the physical layout of the image acquisition environment and the annotation protocols for sub-pixel geometric feature alignment, thereby providing reliable data support for the subsequent training and testing of the vision-geometric fusion architecture, G-VFM.

### 2.1. Experimental Environment and Data Acquisition

**Figure 1:**
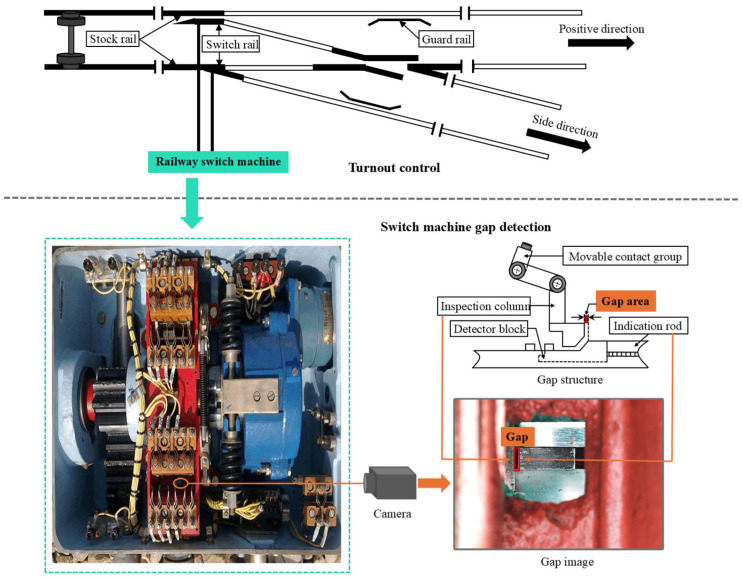
Schematic diagram of the physical layout of the switch machine and the principle of gap detection. The figure shows the macroscopic structure of the railway turnout and the installation position of the switch machine, the internal real-world plan view of the ZD6 switch machine, as well as the gap formation principle based on the check column, the indication rod check block and the actual gap field of view captured by the camera.

To simulate real industrial scenarios, an image acquisition system based on the ZD6 electric switch machine was constructed at a track site. As illustrated in Figure 1, the core monitoring target is the physical gap between the indication rod check block and the check column. The system employs a 16-megapixel industrial-grade autofocus camera, fixed to the interior of the casing’s top cover and using a lateral top-down perspective for observation. The acquisition process encompasses three shooting perspectives and two typical illumination conditions (namely, natural light and low illumination conditions). Meanwhile, a standard feeler gauge was used for the on-site calibration of the physical gaps, acquiring nine groups of high-precision ground truth values ranging from 0.45 mm to 3.05 mm.

### 2.2. Dataset Representation and Annotation Protocol

**Figure 2:**
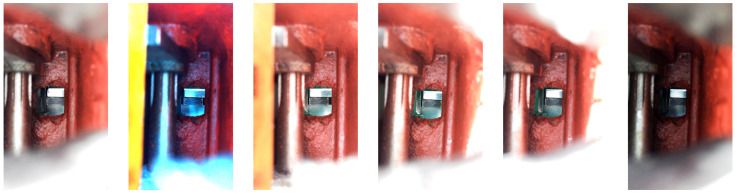
Examples of representative frames from the dataset. From left to right, the images illustrate the imaging effects of various physical sizes ranging from 0.50 mm to 2.50 mm under multi-perspective and variable illumination environments.

This study constructed a dataset comprising 1537 high-definition images with a resolution of 1920 × 1080, which is partitioned into a training set, a validation set, and a testing set at a ratio of 7:1.5:1.5; the specific distribution is shown in Table 1. Figure 2 provides representative sample frames, from which it can be seen that the dataset covers typical gap dimensions ranging from 0.50 mm to 2.50 mm and encompasses multi-perspective and variable-illumination operating conditions. The sample distribution focuses on covering the normal working interval, namely 1.5 ± 0.5 mm, as well as the states near the safety warning boundaries.

**Figure 3:**
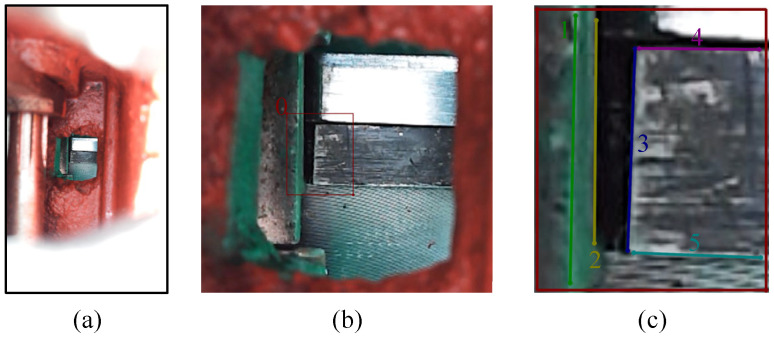
Example of fine-grained annotation. The figure includes (**a**) the original frame, (**b**) the ROI localization annotation shown in red box, and (**c**) the structured point-set annotation of five key geometric edges, corresponding to five line segments in different colors.

To support sub-pixel measurement training, the annotation work adopted a multi-level constraint protocol, with relevant examples shown in Figure 3. To construct supervisory signals with sub-pixel precision, the annotation process follows a cascaded logic of combining macroscopic localization with microscopic representation. First, ROI localization annotation is performed on the key target areas encompassing the check column and the check block to train the YOLOv8 model to achieve anti-disturbance coarse localization under complex operating conditions. Subsequently, under a multi-magnification viewport, high-precision point set annotation is performed on the five core semantic edges within the ROI. By extracting the medial axis features of the edges, a refined topological representation of the physical boundaries is established, thereby providing the data foundation for the subsequent extraction of geometric priors and the correction of visual errors.

## 3. Sub-Pixel Measurement Framework Based on the Fusion of Vision and Geometric Residuals

To address measurement deviations caused by illumination fluctuations, viewpoint shifts, and texture degradation in complex industrial backgrounds, this paper constructed a Visual Geometric Fusion (G-VFM) measurement framework. By combining visual semantic information with physical feature correction, this framework establishes a relatively stable mapping relationship between image sub-pixel features and millimeter-scale physical dimensions.

**Figure 4:**
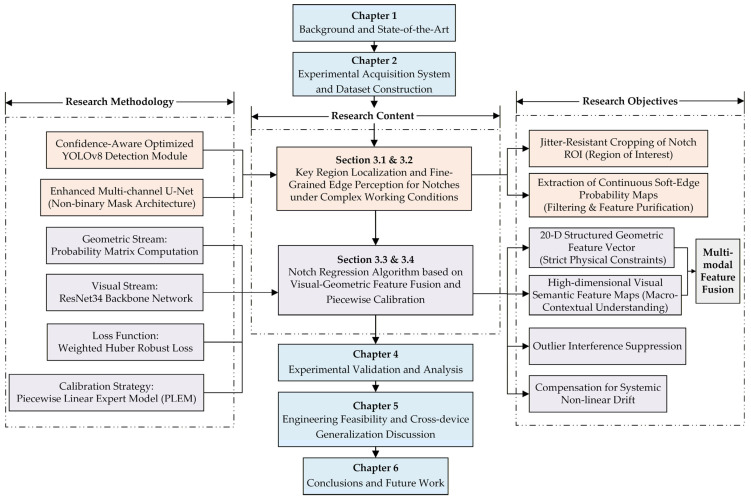
Overall framework and technological roadmap.

As shown in Figure 4, the G-VFM measurement framework adopted in this paper follows a cascaded logic of “localization, perception, and regression,” where each link has a clear division of labor and corresponds to key error sources in gap measurement. Specifically, in the ROI localization stage, an improved YOLOv8 model is used to extract key feature regions from the original image, effectively reducing the impact of background interference and equipment vibration noise through spatial cropping. Subsequently, the process enters the structured edge perception stage. Based on the soft-edge probability maps output by a multi-channel U-Net, a 20-dimensional structured geometric descriptor—containing center position, orientation information, and normal information—is constructed to provide explicit physical constraints for the subsequent regression task. Finally, the system performs fusion modeling of deep visual semantics and geometric priors through an R34-Fusion two-stream residual architecture, and corrects nonlinear drift using a weighted Huber robust loss combined with a piecewise linear calibration strategy, thereby completing the quantitative output of gap dimensions. Overall, the emphasis of this framework lies in constraining visual regression with geometric priors, rather than simply stacking multiple modules, which is the key distinction between the proposed method and conventional visual measurement workflows.

### 3.1. Improved YOLOv8 Localization Strategy

Switch gap dimensions are small and easily affected by complex illumination and background noise. Standard YOLOv8 [42] often faces issues such as insufficient preservation of shallow spatial features and biased confidence outputs when processing such micro-scale targets. To enhance localization stability, this paper introduces staged curriculum training and confidence-aware adjustment strategies while keeping the original Backbone, Neck, and Head topological structures unchanged.

#### 3.1.1. Staged Curriculum Training Strategy

**Figure 5:**
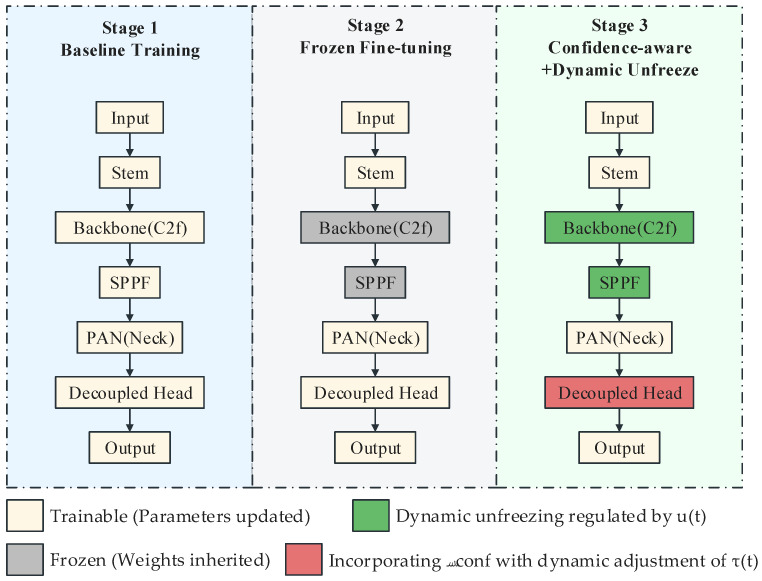
Schematic diagram of the overall topology and staged training strategy of the improved YOLOv8. The figure illustrates the frozen states of parameters and the confidence feedback paths at each stage.

This paper adopts a three-stage progressive training strategy from easy to difficult to balance the preservation of shallow details with high-level semantic adaptation. The overall topology and staged training strategy of the improved YOLOv8 are illustrated in Figure 5. The initial stage is based on pre-trained weights for global parameter updates, enabling the model to possess a stable fundamental detection capability. Subsequently, the process enters the feature solidification stage, where the backbone network parameters are frozen and only the Neck and Head parts are fine-tuned to avoid feature dilution caused by small-sample training and maintain high-resolution spatial information. Finally, in the dynamic perception stage, the backend parameters of the backbone are gradually released as training progresses, and a confidence-aware mechanism is introduced, allowing the model to gradually enhance its ability to capture extremely fine targets along a stable optimization path.

To ensure the reproducibility of the research, Table 2 summarizes the core training parameters for each stage. The parameter settings in the table remain consistent with the experimental section.

#### 3.1.2. Confidence-Aware Loss Optimization

Building upon the baseline detection loss $L_{base}$, this paper further introduces a confidence penalty term $L_{conf}$ to improve the calibration quality of predictions for positive samples. The total loss function $L_{total}$ is defined as:

(1) $$L_{total}=L_{base}+L_{conf},$$

where $L_{base}$ encompasses the box regression and category classification branches, and $L_{conf}$ applies a secondary constraint to positive samples whose predicted confidence ${\hat{c}}_{i}$ is below the target threshold τ:

(2) $$L_{conf}=\lambda \sum_{i\in pos}{\left(\tau -{\hat{c}}_{i}\right)}^{2}\;(\mathrm{if}\;{\hat{c}}_{i}<\tau )$$

The formula, the adjustment coefficient λ is set to 0.5. This mechanism prompts the Objectness output of positive samples to converge toward higher confidence intervals, thereby suppressing pseudo-target interference and enhancing the stability of the non-maximum suppression stage.

#### 3.1.3. Adaptive Threshold Scheduling Mechanism

To make the training process smoother, this paper introduces an adaptive control function $\tau \left(t\right)$ that varies dynamically with training epochs:

(3) $$\tau (t)=\min\left(\tau_{\min}+\Delta \tau \cdot \left\lfloor \frac{t}{k}\right\rfloor ,\tau_{\max}\right).$$

where $\left\lfloor \cdot \right\rfloor$ represents the floor function, ensuring the threshold is updated at fixed intervals of every k epochs, with a step intensity of Δτ=0.02 and an update frequency of k=10. This mechanism adopts a lower threshold in the early training stages to ensure smooth convergence, then gradually increases the constraint intensity to enhance localization precision for millimeter-scale micro-targets while maintaining the integrity of shallow spatial information.

### 3.2. ROI Spatial Alignment and Multi-Channel Edge Perception Model

To eliminate spatial deviations caused by equipment vibration and shooting angles, and to further extract semantic information from fine edges, this paper constructed a high-precision spatial alignment workflow and an improved multi-channel edge perception network.

#### 3.2.1. ROI Extraction and Isotropic Affine Alignment

**Figure 6:**
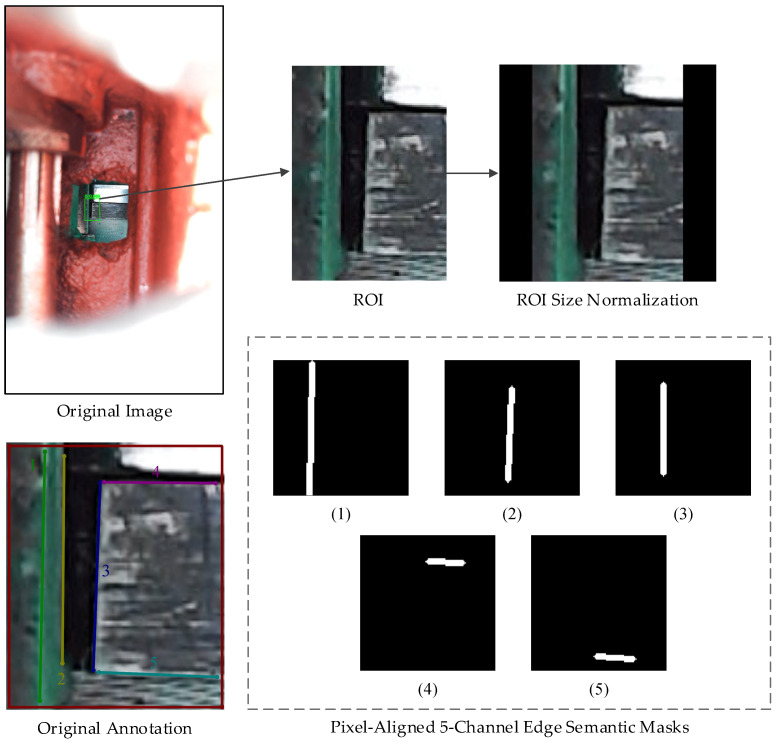
Flowchart of ROI normalization and multi-channel annotation mask generation.

The system crops the target area from the original high-resolution image based on the highest-confidence detection box output by YOLOv8. Figure 6 shows the flowchart of ROI normalization and multi-channel annotation mask generation. To maintain consistency in physical scale between predicted edges and original annotations, an isotropic scaling strategy is adopted for affine transformation. The mapping relationship from original coordinates to the normalized space is defined as:

(4) $$\left[\begin{array}{c} x^{\prime }\\ y^{\prime } \end{array}\right]=s\cdot \left[\begin{array}{c} x\\ y \end{array}\right]-\left[\begin{array}{c} x_{top}\\ y_{left} \end{array}\right]+\left[\begin{array}{c} \Delta x\\ \Delta y \end{array}\right],$$

where s is the scaling factor, $\left(x_{top},y_{left}\right)$ are the coordinates of the ROI’s top-left corner, and $\left(\Delta x,\Delta y\right)$ is the centralized padding offset. This mapping ensures the precise alignment of multi-channel masks and image features within the discrete pixel space, providing the geometric foundation for subsequent sub-pixel calculations.

#### 3.2.2. Improved Multi-Channel U-Net Architecture

**Figure 7:**
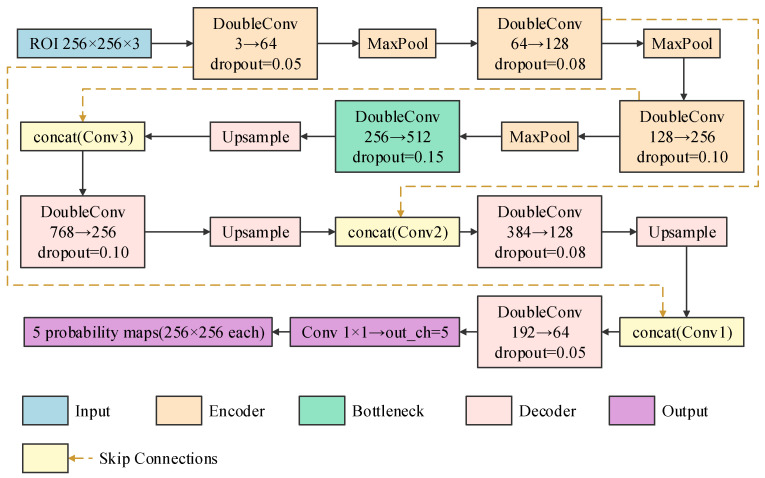
Topology diagram of the improved edge prediction network.

In view of the physical characteristics of switch gap edges being extremely fine and sparsely distributed, this paper purposefully improved the standard U-Net, with the structure shown in Figure 7. The model first decouples multi-task semantics by expanding the output layer into multiple parallel channels to independently predict five key semantic edges, reducing semantic interference between heterogeneous features and improving convergence efficiency in complex textured backgrounds. On this basis, a hierarchical feature suppression mechanism is introduced into the encoder path, using a Dropout probability that increases with network depth, combined with attention enhancement modules at skip connections, allowing the network to actively filter background noise in non-target areas during downsampling. This design, combining decoupling and purification, enhances the perception of high-frequency edge signals while improving representational stability under complex field conditions.

#### 3.2.3. Pixel-Level and Boundary-Aware Hybrid Loss

To simultaneously constrain pixel classification accuracy and edge geometric consistency, a composite loss function $L_{seg}$ was constructed:

(5) $$L_{seg}=L_{WCE}+L_{Dice}+\alpha L_{edge},$$

where the weighted cross-entropy loss $L_{WCE}$ introduces a class-balancing coefficient to compensate for sample imbalance caused by the low proportion of edge pixels; the Dice loss $L_{Dice}$ constrains the overlap between prediction maps and ground truth from a global topological level. To further improve the sharpening capability of sub-pixel boundaries, an edge consistency loss $L_{edge}$ based on the Sobel operator is introduced:

(6) $$L_{edge}=\frac{1}{N}\sum {\left\|\nabla P_{pred}-\nabla M_{gt}\right\|}^{2}.$$

By calculating the gradient residual between the predicted probability map $P_{pred}$ and the mask $M_{gt}$, the model is guided to learn the high-frequency transition features of the edges.

In the training stage, ROI random jitter and morphological enhancement strategies are added to improve generalization. In the inference stage, Gaussian smoothing is used to suppress isolated island noise, combined with a skeletonization algorithm and connected component pruning to ensure the finally extracted single-pixel edges possess good topological continuity, providing high-confidence input for subsequent geometric feature construction.

### 3.3. Structured Geometric Feature Extraction Pipeline and Descriptor Construction

The probability maps output by the edge perception network only contain pixel-level local semantics. To further obtain explicit physical constraints, this paper constructs a 20-dimensional structured geometric descriptor to extract sub-pixel coordinates, topological widths, and orientation information, which serves as a physical prior input for the subsequent regression network.

#### 3.3.1. Dynamic Calibration of the Physical Scale Factor

To establish the mapping relationship between image pixels and physical dimensions, the system utilizes the reference rod channels (edges 4 and 5 in Figure 3c) with known true widths for dynamic scale calibration. Let the edge probability map of the reference channel be $P\left(x,y\right)$, and its column-wise projection distribution be $p\left(x\right)=\sum_{y}P\left(x,y\right)$. After extracting the left and right valid boundaries $\left[x_{left},x_{right}\right]$ of this distribution, the pixel-to-physical scale factor s is calculated as follows:

(7) $$s=\frac{1}{n}\sum_{i=1}^{n}\frac{x_{right,i}-x_{left,i}}{d_{ref}}.$$

This calibration factor is stored synchronously with the sample and is used in subsequent calculations to convert the full-image pixel coordinate system into absolute physical displacement (in millimeters).

#### 3.3.2. Physical Meaning of the 20-Dimensional Geometric Feature Vector

The geometric feature vector $v\in {\mathbb{R}}^{20}$ is composed of independent descriptors from five key semantic edges (edges 1–5 in Figure 3c). A descriptor for each edge contains four core geometric scalars with clear physical meanings:

1. Central abscissa ($\bar{x}$): Represents the sub-pixel horizontal position of the edge in the image coordinate system;
2. Central ordinate ($\bar{y}$): Represents the vertical position of the overall edge distribution;
3. Orientation angle (θ): Represents the tilt posture of the edge in space;
4. Normal projection width (w): The equivalent probability width, representing the overall response intensity and degradation degree of the edge.

The overall 20-dimensional vector structure is defined as:

(8) $$v={\left[\phi^{\left(1\right)},\phi^{\left(2\right)},\phi^{\left(3\right)},\phi^{\left(4\right)},\phi^{\left(5\right)}\right]}^{T}\in {\mathbb{R}}^{20},\;\phi^{\left(c\right)}=\left[{\bar{x}}_{c},{\bar{y}}_{c},\theta_{c},w_{c}\right].$$

This fixed-position encoding method ensures that the topological relationships of multiple types of semantic edges are uniformly constrained within the feature space, thereby guaranteeing the consistency of high-dimensional feature representation.

#### 3.3.3. Sub-Pixel Geometric Quantity Extraction Algorithm

High-frequency noise at industrial sites easily causes edge fragmentation in traditional hard-threshold segmentation. To ensure the stability of the feature extraction pipeline, this paper abandons conventional binarized contour extraction and instead adopts a probability-weighted first-order moment estimation method to obtain continuous coordinates.

First, in the calculation of centroid coordinates $\left(\bar{x},\bar{y}\right)$, the predicted probabilities output by the network are used as the spatial weight distribution to calculate the feature centroid. This method mathematically avoids coordinate jumps caused by discrete binarization and improves robustness against noise interference:

(9) $$\bar{x}=\frac{\sum_{x,y}x\cdot P\left(x,y\right)}{\sum_{x,y}P\left(x,y\right)},\;\bar{y}=\frac{\sum_{x,y}y\cdot P\left(x,y\right)}{\sum_{x,y}P\left(x,y\right)}.$$

Secondly, in the injection of the orientation (θ), considering that the pose of the internal monitoring camera is relatively fixed, the principal imaging direction of the target rod exhibits strong physical stability. Therefore, this paper injects the principal axis of the gap edge, obtained through offline statistics, into the feature vector as a prior constant. This reduces high-variance angular interference caused by local oil contamination, thereby enhancing numerical stability.

Finally, in the calculation of the normal projection width (w), the equivalent geometric width of the target edge is defined by the ratio of the global probability integral to the image height H:

(10) $$w=\frac{1}{H}\sum_{x,y}P\left(x,y\right).$$

#### 3.3.4. Local Environmental Statistics and Feature Standardization

Since cross-condition illumination changes affect measurement precision, the system additionally extracts the mean and standard deviation of the grayscale values in the target ROI and its edge neighborhood, incorporating them into the metadata as environmental state indicators. Subsequently, a Z-score standardization process is applied to the constructed 20-dimensional vector:

(11) $$z_{i}=\frac{v_{i}-\mu_{i}}{\sigma_{i}},$$

where $v_{i}$ is the i-th component in the feature vector, and $\mu_{i}$ and $\sigma_{i}$ are the empirical mean and standard deviation of this component in the training set, respectively. This standardization operation eliminates the dimensional discrepancies among coordinates, angles, and widths, allowing heterogeneous geometric quantities to provide more stable gradient contributions in the subsequent residual fusion network.

### 3.4. Visual Geometric Fusion Regression and Error Calibration

The task of the sub-pixel regression module is to fuse the extracted high-dimensional visual semantics with the structured geometric descriptors and output the final absolute gap dimension. To address the issue where traditional feature concatenation easily leads to low-dimensional geometric information being dominated by high-dimensional visual features, this paper constructed a dual-stream regression architecture, R34-Fusion, that combines a visual baseline with geometric residuals. Simultaneously, targeting the non-uniform distribution and local systematic offsets of field data, a dynamic weighted optimization and a piecewise linear calibration strategy were designed.

#### 3.4.1. Dual-Stream Residual Fusion Architecture and Zero-Initialization Constraint

**Figure 8:**
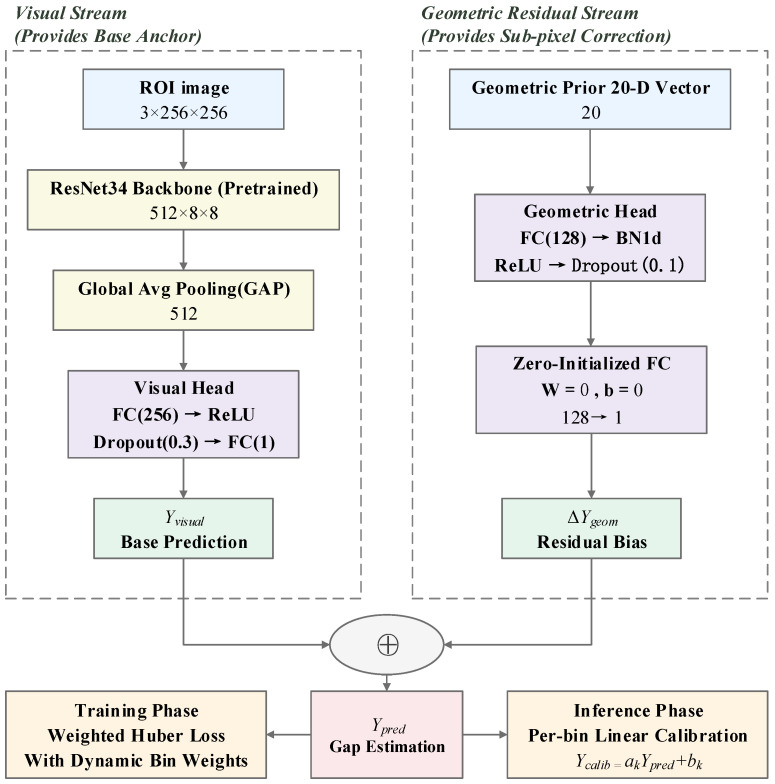
Topology and training/inference flowchart of the R34-Fusion regressor dual-stream architecture.

The R34-Fusion regressor adopts a dual-branch parallel topology to process heterogeneous features; its internal information flow is illustrated in Figure 8. This architecture is synergistically driven by a visual backbone stream and a geometric residual stream. The former relies on a pre-trained ResNet34 to extract multi-scale semantics from the ROI image, maps them into a 512-dimensional feature vector via global average pooling, and outputs the initial baseline value ($Y_{visual}$) of the gap dimension. The latter receives the 20-dimensional structured geometric descriptor and predicts the sub-pixel physical compensation value ($\Delta Y_{geom}$) through a 1D mapping head comprising hidden layers and BatchNorm1d, which is used to perform micro-residual correction on the macroscopic visual prediction using explicit geometric constraints. Ultimately, the system obtains the final predicted value $Y_{pred}$ through terminal linear summation:

(12) $$Y_{pred}=Y_{visual}+\Delta Y_{geom}.$$

To suppress training oscillations during the initial phase of heterogeneous fusion and reduce the negative impact of high-variance noise from geometric features on the visual backbone weights, this paper imposes a zero-initialization constraint on the output layer of the geometric regression head. This design ensures that the model is equivalent to a pure visual baseline network at the beginning of training and gradually activates the geometric branch as iterations progress, thereby achieving a smooth transition from global semantic representation to fine-grained residual compensation.

#### 3.4.2. Dynamic Piecewise Weighting and Huber Robust Loss

Data collected from switch machine sites typically exhibits a non-uniform, long-tail distribution. To prevent normal-state samples from dominating the gradient updates, a sample-level dynamic piecewise weighting mechanism is introduced during the training phase.

The continuous physical label domain is discretized into several key intervals. The unnormalized weight of a sample within a batch is determined by the inverse frequency of its interval and an engineering weight coefficient. The optimization objective employs a weighted Huber robust loss:

(13) $$L_{W-Huber}=\frac{1}{N}\sum_{i=1}^{N}w_{i}\cdot Huber\left(y_{i}-{\hat{y}}_{init,i}\right),$$

where $w_{i}$ is the normalized sample weight within the batch. When the prediction error falls within the micro-error interval (<0.1 mm), the quadratic term of the Huber loss provides stable gradients for fine fitting; when confronting extreme outlier errors, its linear segment effectively truncates the backpropagation of anomalous gradients, thereby enhancing the network’s robustness against environmental noise.

#### 3.4.3. Long-Tail Boundary Optimization and Piecewise Physical Calibration

Addressing the long-tail large-gap data that possesses high early-warning value but scarce samples, such as those greater than 2.5 mm, this paper designed a global-to-local two-stage data reinforcement strategy during the training phase. First, a global oversampling mechanism is introduced during the data loading stage to balance the global optimization gradients by increasing the sampling frequency of tail samples. Subsequently, building upon the global convergence of the backbone network, the tail data subset is extracted, and short-cycle targeted fine-tuning is executed using a lower learning rate to compress the residual variance within the tail’s heterogeneous feature space, while avoiding significant catastrophic forgetting of global features.

Furthermore, although deep regression models possess powerful nonlinear fitting capabilities, they are constrained by the microscopic distortion of the underlying optical imaging system, making it possible that direct prediction results retain local systematic drifts. To this end, a piecewise linear calibration mechanism is cascaded at the end of the inference stage. Specifically, relying on the validation set data, the system independently solves the least-squares fitting equation for each discrete physical interval:

(14) $$Y_{calib}=a_{k}\cdot Y_{pred}+b_{k},$$

where $a_{k}$ and $b_{k}$ represent the sensitivity slope and intercept compensation within that exclusive measurement range interval, respectively. This mechanism mathematically performs piecewise correction of systematic measurement deviations across physical scales, providing a more reliable precision guarantee for the determination of millimeter-level safety warning boundaries.

## 4. Experimental Results and Analysis

This study utilized Python 3.8 and PyTorch 1.11 to construct the algorithmic models. The hardware experimental platform was configured with an Intel Core i7-9750H CPU (2.60 GHz), 16 GB of RAM, and an NVIDIA GeForce RTX 2060 GPU (6 GB). To ensure the consistency of comparison results, all baseline models and ablation models were trained and tested for inference under the same hardware environment.

### 4.1. Evaluation Metrics

To quantitatively evaluate the prediction precision and stability of the G-VFM framework in the gap measurement task, this paper selected Mean Absolute Error (MAE), Root Mean Square Error (RMSE), Maximum Absolute Error (Max Error), and Qualified Rate (QR) as evaluation metrics.

Let the number of samples in the testing set be N, the true physical value of the i-th sample be $y_{i}$, and the model’s predicted value be ${\hat{y}}_{i}$. The definitions and physical significance of each metric are as follows:

1. Mean Absolute Error (MAE): Reflects the overall average degree to which the measured values deviate from the true values, used to measure the system’s measurement accuracy within the global sampling space.
  (15) $$MAE=\frac{1}{N}\sum_{i=1}^{N}\left|y_{i}-{\hat{y}}_{i}\right|.$$
2. Root Mean Square Error (RMSE): Due to its higher penalty weight for large outlier errors, this metric is primarily used to evaluate the dispersion of measurement results and the stability of the system.
  (16) $$RMSE=\sqrt{\frac{1}{N}\sum_{i=1}^{N}{\left(y_{i}-{\hat{y}}_{i}\right)}^{2}}.$$
3. Maximum Absolute Error (Max Error): Records the peak error in the testing set. In the stringent scenarios of railway turnout safety monitoring, this metric is a key boundary parameter for measuring the upper limit of the algorithm’s reliability and “worst-case performance”.
  (17) $$Max\_Error=\underset{1\leq i\leq N}{\max}\left(\left|y_{i}-{\hat{y}}_{i}\right|\right).$$
4. Qualified Rate (QR): Integrating the precision tolerance requirements of railway operation and maintenance, this paper sets an allowable error threshold δ=0.02 mm. If the measurement residual $\left|{\hat{y}}_{i}-y_{i}\right|\leq \delta$, it is determined to be qualified. This metric directly reflects the feasibility of the algorithm in practical engineering delivery. Its definition is:
  (18) $$QR=\frac{1}{N}\sum_{i=1}^{N}I\left(|y_{i}-{\hat{y}}_{i}|\;<\;\delta \right),$$

where $I\left(\cdot \right)$ is an indicator function that takes the value 1 when the condition in the parentheses holds, and 0 otherwise.

### 4.2. Performance Verification of the Preprocessing Module

The preprocessing module includes ROI localization and edge extraction, and its output quality directly determines the upper limit of input precision for the subsequent feature fusion and regression stages. This section evaluates the effectiveness of the improvement strategies through ablation experiments in terms of spatiotemporal consistency and geometric topological purity.

#### 4.2.1. ROI Localization Stability Analysis

**Figure 9:**
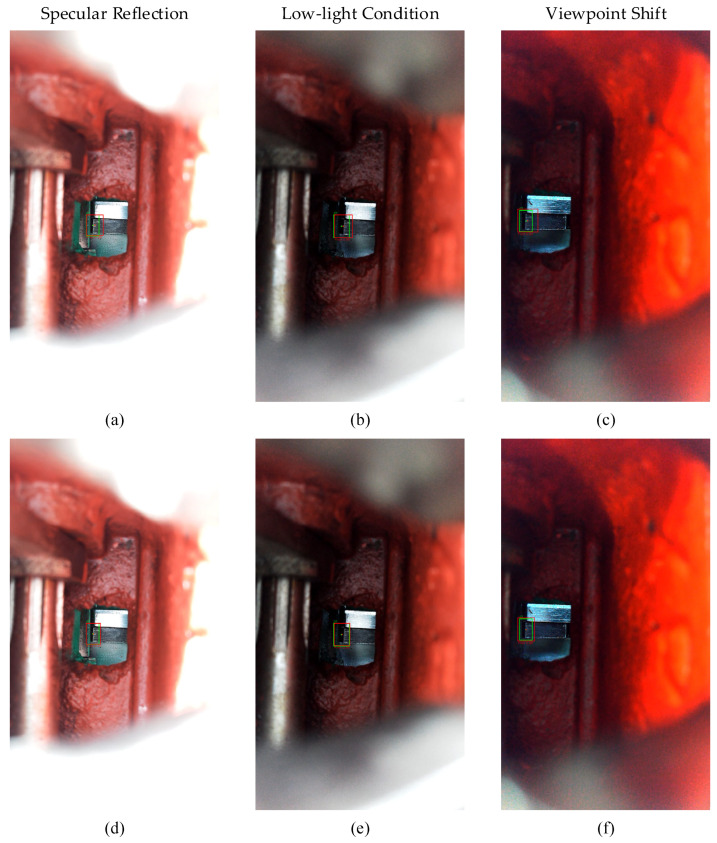
Comparison of ROI localization stability under typical operating conditions. The first row (**a**–**c**) shows the baseline model results; in strong reflection and low-light scenarios, the predicted boxes (red) exhibit obvious spatial deviation from the ground truth (green). The second row (**d**–**f**) shows the improved model results, which maintain precise alignment even under disturbances such as viewpoint shifts.

Considering that on-site railway monitoring equipment is usually in a high-frequency vibration environment, the inter-frame stability of ROI localization holds more engineering significance than the mere detection rate. If the localization box jitters significantly, it will introduce non-physical coordinate noise, which in turn interferes with the construction of geometric feature vectors. To this end, this paper compared the performance of the improved YOLOv8 with the baseline model YOLOv8-Baseline on 229 test samples, and introduced Center Shift as a stability evaluation metric. The experimental results are presented in Figure 9 and Table 3.

The experimental results show that the baseline model exhibits significant localization uncertainty under blurred boundaries and complex illumination conditions, with an average center drift reaching 27.07 pixels. Thanks to the confidence-aware adjustment and staged training strategies, the improved model reduced the average shift to 7.05 pixels, which accounts for about 0.3% at 1080p resolution, indicating that this strategy effectively suppresses pseudo-errors caused by coordinate jitter and provides a more stable spatial reference for subsequent geometric feature extraction.

#### 4.2.2. Quality Assessment of Fine-Grained Edge Extraction

High-quality edge masks are the foundation for constructing geometric descriptors. To evaluate the quality of edge extraction, this paper compared the performance of the improved U-Net, the baseline U-Net, and the traditional Canny operator in complex environments. The experimental results are presented in Table 4. As precision measurement tasks are very sensitive to the position of the edge centerline, relying solely on the Dice coefficient makes it difficult to accurately describe minute shifts in edges; therefore, this paper introduces skeleton-based evaluation metrics. S-Dice is used to measure the degree of topological overlap between the extracted skeleton and the true skeleton, while S-HD95 is used to characterize outlier noise and edge burrs between skeletons, thereby forming a comprehensive evaluation system from topological structure to spatial deviation.

Qualitative results show that traditional edge operators perform poorly in complex environments. Canny generates a large number of pseudo-edges under heavy oil pollution and strong reflection conditions, with an S-Dice of only 0.0520, which can no longer support subsequent geometric calculations. Although the baseline U-Net is significantly better than Canny in terms of overall overlap, because it is constrained by a single cross-entropy loss, its output probability distribution is too smooth when processing parallel edges at extremely close distances, which easily causes topological adhesion and thus weakens the identification ability of physical gaps. In contrast, by introducing the Sobel edge loss, the improved model forms a clearer probability transition zone between extremely close edges, helping maintain the independence of multiple edges. The experimental results show that the S-HD95 of the improved model is further reduced, indicating that it has better performance in suppressing burrs and maintaining topological continuity, laying the foundation for subsequent sub-pixel geometric feature extraction.

**Figure 10:**
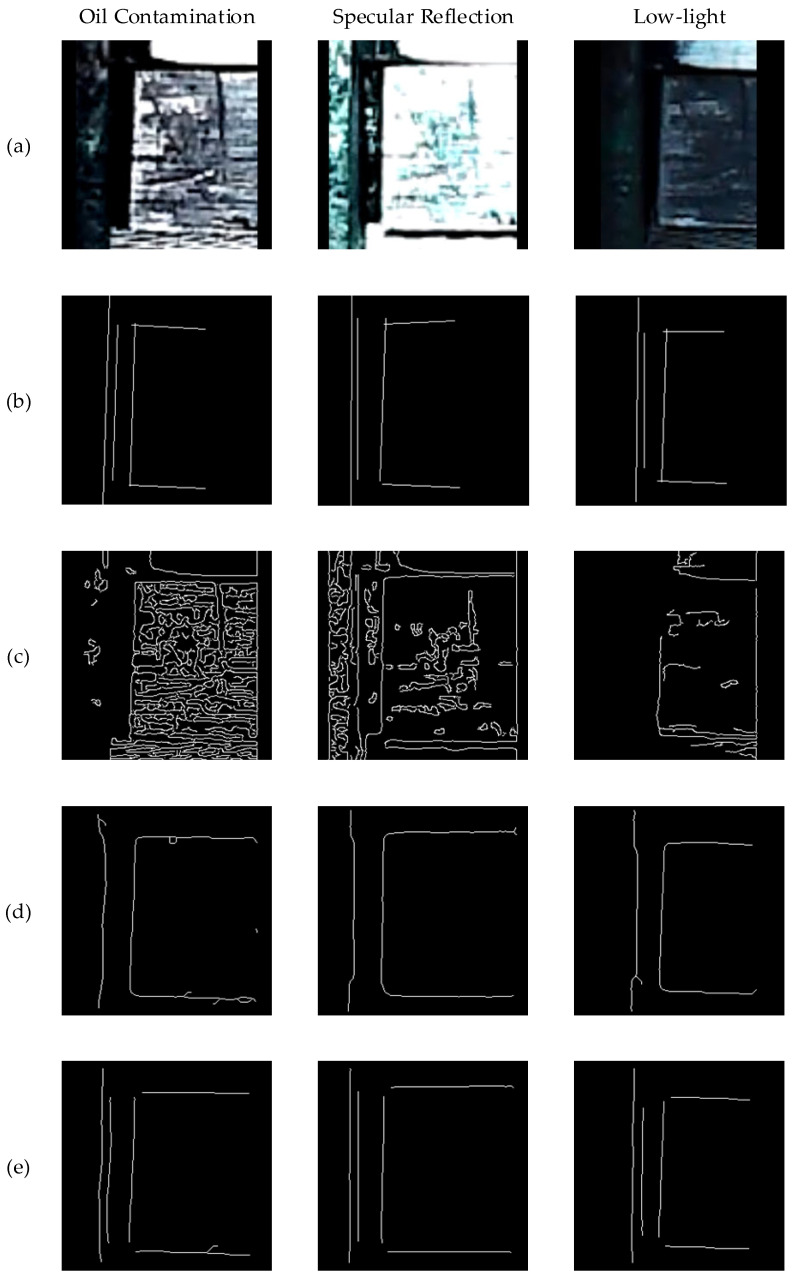
Qualitative quality comparison matrix of different edge extraction methods. The five rows from top to bottom are: (**a**) Original ROI images; (**b**) Manual annotation ground truth; (**c**) Canny operator detection results; (**d**) Baseline U-Net prediction results; (**e**) Improved U-Net prediction results of this paper.

Figure 10 shows the comparison results of different edge extraction methods under heavy oil pollution, metal reflection, and low-light conditions. It can be seen that while suppressing background noise, the improved model can well maintain the continuous structure of single-pixel-level edges.

### 4.3. Core Method Comparison and Sub-Pixel Mechanism Analysis

#### 4.3.1. Quantitative Accuracy Evaluation

The previous sections verified the topological extraction capability of the preprocessing module; however, in precision engineering measurement, semantic coherence does not equate to measurement accuracy. This section aims to verify the necessity and efficacy of the proposed visual and geometric residual fusion regression architecture under a 0.02 mm industrial tolerance through quantitative comparison and error mechanism analysis. Considering the current lack of a unified sub-pixel level open-source benchmark in the field of switch machine gap monitoring, this paper selects representative mainstream methods from the fields of visual measurement and deep learning for full-range comparative testing. These methods are categorized into four types: traditional geometric methods, segmentation-centroid methods, end-to-end sub-pixel regression methods, and the proposed method. The relevant performance metrics are shown in Table 5.

The experimental results indicate significant differences among various paradigms when dealing with stringent industrial-grade tolerances. Segmentation-based methods, while achieving good regional coverage, are essentially discrete classifications on a pixel grid. Consequently, their measurement accuracy is constrained by the spatial resolution of the image, making it difficult to break through to the sub-pixel level. Accordingly, the MAE of Unet++, DeepLabv3+, and the improved U-Net remains around 0.25 mm with low QR, suggesting that these methods are more suitable as structural extraction tools rather than for direct precision dimension regression tasks.

The end-to-end regression methods significantly outperform the segmentation methods in overall accuracy. Among them, ResNet50 and Swin-Transformer have managed to compress errors to a low level; however, they still exhibit obvious peak error fluctuations under complex backgrounds and sparse sample conditions, with Max Errors exceeding 0.05 mm. This indicates that in the absence of explicit geometric constraints, the stability of these models on long-tail samples remains limited. In contrast, the proposed G-VFM compresses the global MAE to 0.0076 mm and keeps the maximum absolute error within 0.0193 mm, making it the only model in the table to fall completely within the 0.02 mm tolerance boundary. This demonstrates that joint modeling of visual semantics, geometric residual streams, and piecewise calibration contributes to improving the consistency of measurement results across the entire range.

#### 4.3.2. Sub-Pixel Advantage and Mapping Consistency Analysis

To further analyze the mechanism by which the proposed regression architecture breaks the pixel grid limitation, this study selected the improved U-Net probability map coupled with the intensity-centroid approximation method as a control group and compared its predictive behavior with the proposed method across a continuous physical range.

**Figure 11:**
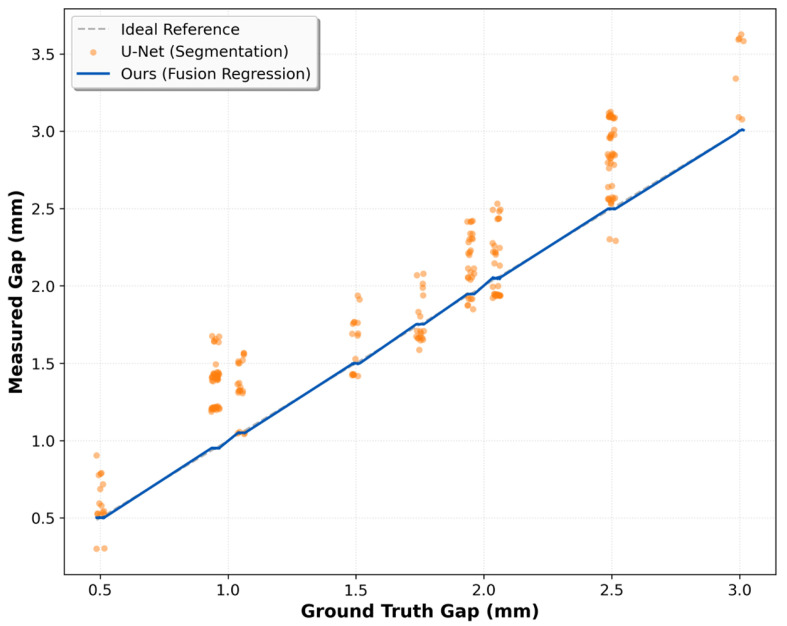
Analysis of measurement consistency and sub-pixel advantages. The orange scatter points represent the method based on the improved U-Net probability map supplemented by intensity-centroid approximation; the blue solid line represents the results of the proposed regression architecture.

Figure 11 shows that the segmentation-based method exhibits a prominent stepped discrete distribution within local intervals. The root cause of this phenomenon is that the semantic segmentation task is essentially a pixel-level classification problem; when the true physical displacement change is smaller than the physical equivalent of a single pixel, the output tends to stay near the discrete grid, forming a zero-order hold state. Therefore, relying solely on segmentation maps and centroid estimation makes it difficult to further improve measurement continuity and boundary stability.

In contrast, the proposed method exhibits a relatively smooth response curve across the entire range and maintains high consistency with the true reference line. Benefiting from the guidance of the explicit geometric feature stream, the regression fusion architecture maps the prediction space from the discrete pixel domain to the continuous physical real-number domain, effectively neutralizing the stepped discrete noise. Even compared to regression methods like ResNet50, the proposed method maintains better stability in critical micro-change intervals, indicating higher representation fidelity in precision measurement tasks.

### 4.4. Ablation Study of Key Modules

**Figure 12:**
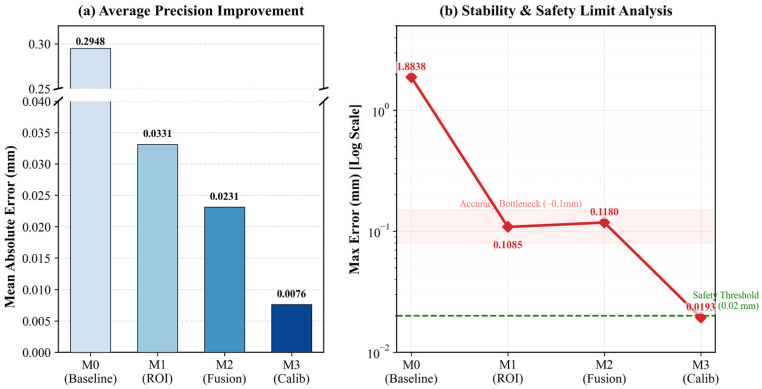
Performance evolution analysis of the ablation study. The figure shows the trend of MAE as modules are introduced and the distribution range of Max Error.

To decouple the marginal contributions of each core module to the system performance, this study designed four progressive ablation models. M0 is a baseline model that performs end-to-end regression directly on full-resolution images without spatial attention mechanisms or prior feature injection. M1 introduces ROI cropping on this basis to weaken background noise through spatial domain decoupling. M2 further adds the visual and geometric residual fusion branch, allowing the 20-dimensional structured features to participate in correcting the visual backbone’s predictions. M3 then adds a piecewise linear calibration module to the output of M2 to compensate for systematic deviations across discrete physical range intervals. The quantitative results for each model are summarized in Table 6, and the corresponding error evolution trends are illustrated in Figure 12.

The experimental results show that the error of M0 is relatively high, indicating that unconstrained full-image regression is susceptible to significant interference from industrial on-site noise. After introducing ROI cropping, the MAE of M1 drops significantly, proving that pre-spatial decoupling plays a fundamental role in improving the signal-to-noise ratio of features. With the further addition of the geometric residual stream, the overall accuracy of M2 continues to improve, demonstrating that explicit geometric priors can effectively alleviate the representation degradation of pure visual networks in weak-texture regions. However, the maximum error of M2 is slightly higher than that of M1, suggesting that pure data-driven residual fusion still struggles to completely eliminate extreme deviations caused by long-tail samples. Finally, with the introduction of piecewise linear calibration, M3 compresses both the average and peak errors to low levels, becoming the only model to fully meet the 0.02 mm tolerance requirement. This explains that in high-precision industrial measurement tasks, there are limits to relying solely on deep feature fitting, and posterior calibration remains of practical significance for suppressing residual systematic biases.

### 4.5. System Error Analysis and Piecewise Calibration Mechanism

Constrained by operational specifications at industrial sites, it is often difficult to acquire annotated data covering a continuous full-scale range. The dataset constructed in this study is based on 10 key state nodes of railway maintenance, exhibiting significant discrete and sparse distribution characteristics. Under these conditions, the prediction deviation of the fusion model M2 tends to exhibit local heterogeneity. Therefore, it is necessary to further analyze the necessity and error correction mechanism of the M3 piecewise linear calibration.

#### 4.5.1. Calibration Protocol and Comparative Evaluation

To ensure transparency in the calibration process and fairness in evaluation, this paper establishes an offline calibration protocol. Based on the physical states of switch machine operation and maintenance, 0.50, 0.95, 1.05, 1.50, 1.75, 1.95, 2.05, 2.50, 3.00, and 3.30 mm are designated as the centers of 10 physical segments. The boundaries of these intervals are determined by the midpoints between adjacent centers to achieve continuous full-scale coverage. The calibration process exclusively utilizes independent validation set data and executes least-squares fitting within each interval to obtain local correction coefficients (slope $a_{k}$ and intercept $b_{k}$). To avoid local fitting instability caused by insufficient samples, a fallback logic is implemented: when the number of samples in a certain interval is less than 5, the system automatically replaces local parameters with global linear parameters. Once calibration is complete, all coefficients are applied to the testing phase in the form of a static lookup table, ensuring the independence and consistency of the evaluation process.

This paper performs a full-range comparison between the piecewise calibration strategy (M3) and global linear regression, cubic polynomial regression, and non-parametric isotonic regression. The comparison results are presented in Table 7. The results show that global linear and polynomial regression have limited effects on correcting residual errors, indicating that the system bias is not caused by a single low-order distortion, but rather presents local differences that vary with the physical scale. While Isotonic Regression can reduce the average error, its peak error remains significantly higher than the safety threshold, suggesting that pure data-driven non-parametric methods are prone to over-smoothing on sparse nodes. In contrast, M3 not only achieves the lowest average error but also controls the maximum error within 0.02 mm, proving that local linear mapping based on physical intervals is more suitable for sparse monitoring scenarios.

#### 4.5.2. Prediction Residual Distribution and Mechanism Analysis

**Figure 13:**
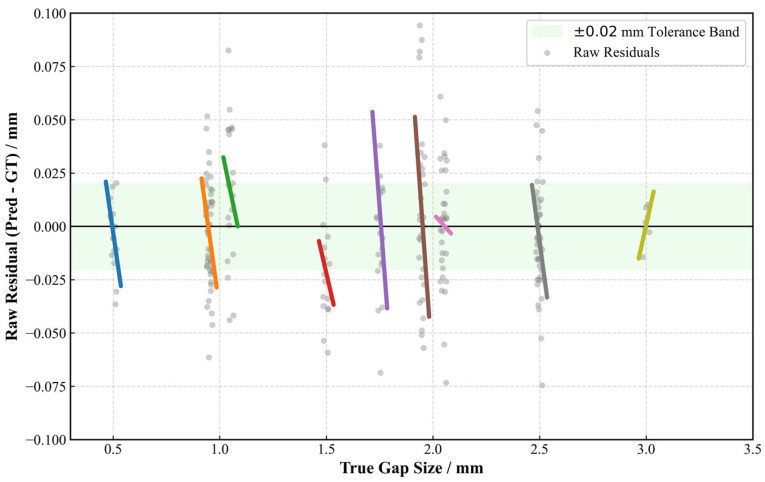
Prediction residual distribution and piecewise calibration mechanism analysis. Gray scatter points reflect random variance, and colored solid lines represent the local linear fitting trajectories of M3 within each independent interval.

**Figure 14:**
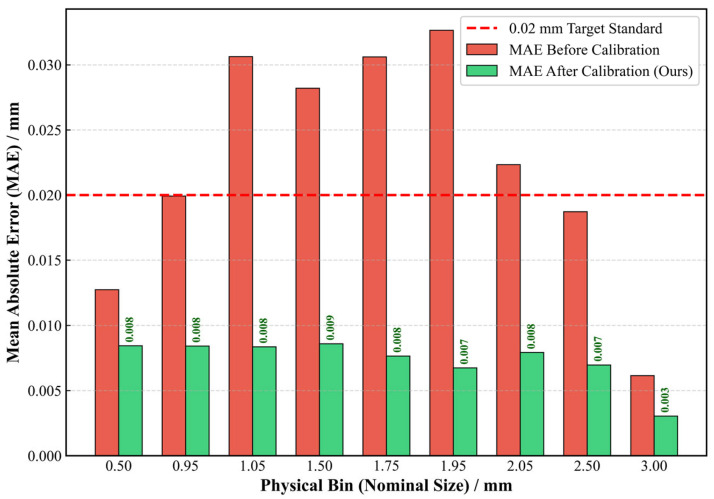
Comparison of MAE at each key node before and after calibration.

To further explain the calibration mechanism of M3, this paper visually analyzes the original residual distribution and the local errors before and after calibration, as shown in Figure 13 and Figure 14. The gray scatter points in the figure reflect the distribution of random variance, while the colored solid lines represent the local linear fitting trajectories of M3 within each independent interval.

The performance improvement of M3 primarily stems from the differentiated treatment of random variance and systematic bias. Figure 13 shows that the prediction residuals exhibit significant non-uniformity across different physical ranges, indicating that sensitivity and offset vary at different scales—this is the reason why global models struggle to achieve stable performance. The piecewise strategy transforms complex nonlinear drifts into multiple local linear relationships, giving the error compensation a clearer physical correspondence.

At the engineering application level, piecewise calibration is particularly important for the reconstruction of critical boundary states. Taking the 1.05 mm warning node as an example, the uncalibrated model is prone to determination ambiguity due to local bias. After correction by M3, the local residuals at each node are further compressed (Figure 14), and the maximum error is controlled within a low range, thereby reducing the probability of false alarms and missed detections.

### 4.6. System Robustness and Measurement Reliability Evaluation Under Complex Operating Conditions

Railway switch machine gap monitoring systems are typically deployed long-term in complex environments such as outdoors or in tunnels. Drastic illumination changes, strong reflections, and sensor thermal noise may all affect system stability. At the same time, high-precision measurement systems need to maintain repetitive stability under different times and environmental fluctuations. To demarcate the performance boundary and confidence level of the proposed measurement architecture in actual engineering deployment, this section first constructs a stress test subset containing illumination perturbations and signal degradation based on a physical degradation model to evaluate the representation stability of the model under extreme conditions. Subsequently, through multi-batch cross-validation and uncertainty evaluation, the measurement reliability of the system in normal operation is further demonstrated.

**Figure 15:**
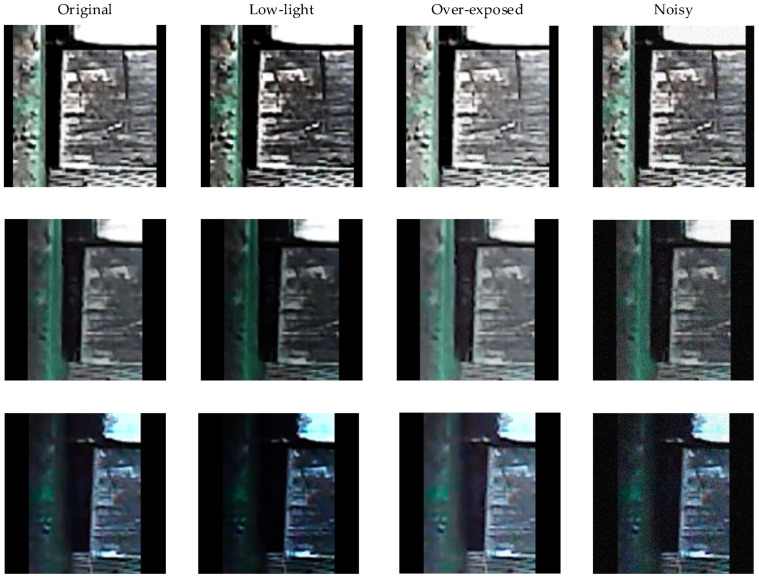
Visual degradation matrix under complex operating conditions. The figure displays the imaging states of three typical gap samples under shadow engulfment, local over-exposure, and high-frequency noise coverage.

Figure 15 illustrates the specific simulated interference scenarios. By applying a Gamma transformation with γ=1.5 to compress the dynamic range of the dark regions, the system simulates low-contrast imaging conditions typically found deep in tunnels or in environments lacking supplementary lighting, thereby evaluating the model’s feature-retention capability in weak-signal backgrounds. To address pixel saturation caused by direct strong light or specular reflections on metal surfaces, a non-linear transformation with γ=0.7 is utilized to simulate local texture loss, examining the model’s regression stability when critical information is compromised. Furthermore, to simulate photosensitive chip thermal noise or long-cable transmission interference induced by extreme temperature differences, Gaussian white noise with σ=0.005 is injected into the original images. These multi-dimensional degradation experiments, spanning the dimensions of illuminance extremes and signal quality, provide a controlled testing environment for evaluating the algorithm’s reliability under complex field conditions.

The calibrated M3 model was directly deployed on the degradation dataset for blind testing, during which all network parameters and calibration operators were frozen. The results are summarized in Table 8.

The results show that the system has small performance fluctuations under Gaussian noise, with the MAE only slightly increasing from 0.0076 mm to 0.0078 mm and the qualified rate remaining at 99.6%, indicating strong suppression capability against random noise. Under over-exposure and low-light conditions, the MAEs are 0.0084 mm and 0.0100 mm, with qualified rates of 96.1% and 94.8%, respectively, demonstrating a certain adaptability to illumination changes, although significant degradation still occurs under extremely low-light conditions. The system’s stability primarily stems from the front-end edge perception module’s noise suppression capability and the smoothing effect formed by the backbone residual network during the multi-scale feature extraction process. In over-exposed and dark scenes, as long as the edge topology has not completely disappeared, the geometric residual stream can still constrain the spatial mapping relationship to a certain extent, preventing the overall failure often seen in traditional pure vision methods due to pixel intensity mismatch.

However, stress testing also indicates clear performance boundaries for the system. Especially under extremely low-light conditions, the maximum error rises to 0.0767 mm, indicating that when the dynamic range of dark regions is excessively compressed, leading to significant truncation of edge pixels, the underlying geometric structure will suffer irrecoverable loss—a loss that exceeds the correction range of the piecewise linear calibration module. In other words, while the proposed method maintains good measurement stability in most complex scenarios, its robustness is still predicated on the basic discernibility of the edge topology. To further enhance adaptability in actual engineering deployment, hardware compensation measures such as active lighting or multi-source sensing should be considered in the future to mitigate the impact of extreme environments on visual measurement.

To further verify the repeatability and measurement reliability of the system, this paper conducted multi-batch cross-validation and measurement uncertainty evaluation in a normal baseline environment. Specifically, the testing set, which includes real measurement conditions, was divided into 6 independent evaluation batches according to the collection time sequence and environmental changes. The statistical results for each batch are shown in Table 9. It can be seen that the MAE across different batches ranges from 0.0056 mm to 0.0090 mm with small fluctuations; the standard deviation of MAE among the 6 batches is only 0.0012 mm, indicating stable output consistency of the method across different collection batches. Further, according to the specifications for expressing measurement uncertainty, an uncertainty evaluation was performed on the entire testing set. The results show that the Type A standard uncertainty of a single measurement for the system $\mu_{A}$ is 0.0090 mm. Under the condition of a coverage factor k=2 (corresponding to approximately 95% confidence probability), the expanded uncertainty $U=k\cdot u_{A}=0.0179\;\mathrm{mm}$ remains at a low level, indicating that the statistical fluctuations of the system can be generally controlled within the 0.02 mm industrial tolerance range, providing reliable precision support for field applications.

## 5. Discussion

This chapter further analyzes the experimental results from two aspects: engineering deployment feasibility and cross-device transfer capability. The former focuses on the computational load, operational rhythm, and maintenance conditions of the algorithm at actual railway sites, while the latter discusses the applicability and adaptation methods of the proposed method on other models of switch machines.

### 5.1. Computational Overhead and Actual Deployment Evaluation

In industrial precision monitoring scenarios, inference efficiency is a key indicator for measuring the engineering feasibility of an algorithm. Since G-VFM adopts a multi-stage cascaded structure involving ROI localization, edge extraction, geometric feature construction, and residual regression, it is necessary to examine whether its computational overhead meets the near-real-time processing requirements of railway sites. Based on an Intel Core i7-9750H CPU and an NVIDIA GeForce RTX 2060 GPU platform, this paper collected statistics on the complete process of a single 1080p image from input to the final physical dimension output. The results show that the average end-to-end inference latency of the system is 52.23 ms, corresponding to a processing speed of 19.15 FPS.

Combining this with the operational rhythm of the switch machine, the typical action cycle of a ZD6 switch machine is about 3 to 5 s, and gap measurement is essentially a quasi-static monitoring task performed after the turnout switch is completed. Therefore, the processing speed of 19.15 FPS can meet the timeliness requirements of a single measurement and can also adapt to the continuous inspection needs under edge computing nodes. From this perspective, the computational overhead of the system is within an acceptable range, laying the foundation for further deployment.

To more closely reflect the actual railway service environment, this paper also comprehensively evaluated several engineering factors in system deployment. First, regarding camera stability, railway turnouts are subject to strong high-frequency vibrations when trains pass. Although the front-end YOLOv8 has a certain dynamic ROI cropping capability that can mitigate the impact of slight field-of-view shifts, highly rigid anti-vibration brackets should still be configured during actual installation to reduce the interference of motion blur on imaging quality. Second, regarding recalibration frequency, the piecewise linear calibration mechanism adopted by M3 can well correct systematic errors; however, as the equipment serves over the long term, bracket fatigue and lens aging may still introduce slowly accumulating deviations. Therefore, it is recommended to periodically recheck the calibration baseline in conjunction with the routine maintenance cycles of railway engineering departments to maintain the stability of measurement results. Third, regarding maintenance needs, the routine maintenance of the system mainly focuses on the cleaning of front-end optical lenses and the thermal management of edge computing units, which possess strong practical necessity in trackside environments.

Synthesizing the above results, it can be seen that G-VFM, without any model quantization or compression, has already achieved a good balance between accuracy and efficiency, and can adapt to the basic operational rhythm of railway maintenance. Given the current experimental conditions, this method possesses certain engineering application potential, but its deployment effectiveness remains closely related to on-site installation conditions and maintenance levels.

### 5.2. Algorithm Generalization Discussion and Cross-Model Transfer Analysis

Regarding the issue of cross-device generalization, it must be clarified that although the proposed G-VFM framework has been verified on the ZD6 switch machine dataset, its underlying process follows the general logic of localization, segmentatio n, geometric construction, and fusion regression, and thus possesses a certain foundation for transfer structurally. However, this transfer capability does not imply that it can be directly generalized to other equipment models without modification. Especially when there are differences in mechanical topology, reference plane positions, and gap definitions, relevant modules still require targeted adaptation.

Taking the ZYJ7 and S700K switch machines as examples, these two types of equipment differ significantly from the ZD6 in driving methods and gap indication mechanisms. Consequently, the front-end object localization network and edge perception network typically need to relearn the corresponding target features, and the definition of geometric priors must also be adjusted according to changes in the mechanical structure. If the proposed model is completely reused without reconstruction, it is often difficult to ensure the physical consistency of geometric descriptors. A more robust approach is to adopt a transfer learning strategy based on the existing feature extraction layers, fine-tune the front-end modules using a small number of samples from the target model, and then redefine the calculation method of geometric residuals according to the mechanical drawings of the new machine. This approach not only retains the existing representation capabilities of the deep visual networks but also allows geometric priors to continue exerting physical constraints, thereby improving the interpretability and operability of cross-model transfer.

It should also be pointed out that although the dataset used in this paper covers various illumination and noise conditions, it still belongs to the category of controlled collection as a whole and is primarily constructed based on ZD6 equipment. Therefore, the current results are more suitable for demonstrating that the method has good stability within the covered operating conditions, rather than being directly extrapolated as a universal conclusion applicable to all switch machine models and environmental conditions. If subsequent research aims to further expand the application scope, it will be necessary to construct cross-model joint datasets and continue verifying its representation stability and error control capability on equipment with greater mechanical structure differences.

## 6. Conclusions and Prospect

This paper proposes and verifies a visual and geometric residual fusion gap measurement framework (G-VFM) to address the challenges of high sub-pixel accuracy requirements, strong on-site interference, and insufficient stability of traditional visual methods in switch machine gap monitoring. By cascading localization and semantic segmentation modules, the framework achieves relatively stable single-pixel edge extraction under complex operating conditions, providing a more reliable topological foundation for subsequent geometric feature analysis. The R34-Fusion network further integrates macroscopic visual textures with microscopic geometric residuals, alleviating the quantization step effect common in traditional segmentation methods to a certain extent. This enables the measurement process to gradually transition from discrete pixel classification to continuous physical dimension regression, thereby enhancing the dimensional representation capability at the sub-pixel scale.

Experimental results indicate that, combined with the piecewise linear calibration mechanism, the G-VFM system compresses the full-range MAE to 0.0076 mm and controls the maximum absolute error within 0.0193 mm, achieving a 100% qualified rate under the 0.02 mm industrial tolerance requirement. The system’s end-to-end inference time is 52.23 ms, corresponding to 19.15 FPS, indicating that under the current experimental conditions, the method balances relatively high measurement accuracy with good processing efficiency, meeting the basic requirements of near-real-time industrial inspection. Further tests under complex conditions and cross-batch analysis also show that the method exhibits stable output performance across the covered degradation scenarios and collection conditions; however, its performance still experiences some degradation under extremely low-light conditions and obvious structural information loss.

Nevertheless, the work in this paper still has certain limitations. First, the experimental data are mainly derived from ZD6 switch machines; thus, the current conclusions are more applicable to this model and its similar scenarios, and the cross-device generalization capability remains to be further verified. Second, this paper has not yet conducted direct testing under stronger dynamic vibration conditions; therefore, the stability performance in high-frequency, strong-vibration environments still needs to be further analyzed in conjunction with more representative on-site disturbance scenarios. Subsequent research will continue to focus on model lightweighting, cross-model transfer, and stability improvement under complex dynamic interference conditions. We will attempt to further expand the application scope of this method to similar equipment and more complex operating conditions through means such as transfer learning, feature adaptation, and temporal constraints.

## Figures and Tables

**Table 1:** Size distribution and partitioning of the ZD6 switch machine gap dataset.

Gap Category	Gap Size (mm)	Total Samples	Training Set	Validation Set	Testing Set
1	0.50	108	76	16	16
2	0.95	302	211	45	46
3	1.05	135	95	20	20
4	1.50	111	78	17	16
5	1.75	132	94	19	19
6	1.95	194	136	29	29
7	2.05	208	146	31	31
8	2.50	297	207	44	46
9	3.00	50	36	8	6
Total	-	1537	1079	229	229

**Table 2:** Core parameter configuration for each training stage of the improved algorithm.

Parameter Name	Symbol	Stage I	Stage II	Stage III
Initial Learning Rate	$lr_{0}$	0.0001	0.0005	0.001
Weight Decay	wd	0.0005	0.0005	0.0001
Confidence Adjustment Coefficient	λ	-	-	0.5
Initial Confidence Threshold	$\tau_{min}$	-	0.85 (Fixed)	0.90
Terminal Confidence Threshold	$\tau_{max}$	-	-	0.95

**Table 3:** Quantitative comparison of ROI localization stability.

Method	Detection Rate	Avg Center Shift (px)	Max Center Shift (px)	Stability Assessment
YOLOv8 (Baseline)	100.0%	27.07	1090.30	Unstable
Ours (Improved)	100.0%	7.05	21.42	High Stability

**Table 4:** Comparison of skeleton-based edge extraction precision.

Method	S-Dice	S-HD95 (px)	Topological Quality
Canny	0.0520	140.62	Discontinuous/Noisy
U-Net (Baseline)	0.8478	22.62	Jagged/Artifacts
Ours (Improved)	0.8473	19.86	Smooth/Clean

**Table 5:** Comparison of performance metrics across different measurement strategies.

Technical Paradigm	Core Model (Baselines)	MAE (mm)	RMSE (mm)	Max Error (mm)	Qualified Rate (QR < 0.02 mm)
Traditional	Canny	2.3149	3.1036	6.0927	0.0%
Segmentation	Unet++	0.2672	0.3232	0.6841	3.49%
	DeepLabv3+	0.2493	0.3051	0.6927	3.49%
	Improved U-Net	0.2827	0.3396	0.7367	2.6%
Regression	ResNet50	0.0136	0.0175	0.0524	77.73%
	Swin-Transformer	0.0190	0.0238	0.0669	59.83%
Proposed	Ours G-VFM	0.0076	0.0089	0.0193	100.0%

**Table 6:** Quantitative results of the ablation study for key modules.

Model ID	Configuration	MAE (mm)	RMSE (mm)	Max Error (mm)	Relative Improvement (vs. Prev)
M0	Baseline (Full Image)	0.2948	0.4526	1.8838	-
M1	+ROI Cropping	0.0331	0.0419	0.1085	+88.8%
M2	+Geometric Residual Fusion	0.0231	0.0308	0.1180	+30.2%
M3	+Piecewise Calibration	0.0076	0.0089	0.0193	+67.1%

(Note: Relative improvement in MAE refers to the degree of improvement compared to the previous stage’s model configuration).

**Table 7:** Performance comparison of different calibration strategies.

Calibration Method	Type	MAE (mm)	RMSE (mm)	Max Error (mm)	Safety Check (<0.02 mm)
M2 (Raw)	Uncalibrated	0.0231	0.0308	0.1180	Fail
Global Linear	Parametric	0.0231	0.0308	0.1183	Fail
Polynomial (Deg = 3)	Parametric	0.0235	0.0311	0.1192	Fail
Isotonic (SOTA)	Non-parametric	0.0094	0.0147	0.0836	Fail
Ours M3 (Per-bin)	Physics-aware	0.0076	0.0089	0.0193	Pass

**Table 8:** System robustness test results under complex operating conditions.

Test Condition	Physical Scenario Simulation	MAE (mm)	Max Error (mm)	Qualified Rate (QR < 0.02 mm)
Standard (Clean)	Baseline environment	0.0076	0.0193	100.0%
Sensor Noise	Gaussian thermal noise	0.0078	0.0232	99.6%
Over-exposure	Strong light/metal reflection	0.0084	0.0292	96.1%
Low-light	Nighttime/shadows	0.0100	0.0767	94.8%

**Table 9:** System repeatability verification and uncertainty analysis across collection batches.

Collection Batch (Session)	Sample Count	MAE (mm)	Std. Dev (mm)	Max Error (mm)
Session 1	57	0.0082	0.0044	0.0164
Session 2	25	0.0090	0.0040	0.0166
Session 3	56	0.0073	0.0046	0.0177
Session 4	42	0.0070	0.0046	0.0193
Session 5	24	0.0056	0.0050	0.0164
Session 6	25	0.0087	0.0054	0.0162
Overall Statistics	229	0.0076	--	0.0193

## Data Availability

The data presented in this study are available on request from the corresponding author. The data are not publicly available due to privacy restrictions related to industrial railway deployment.
